# Production of bioherbicide by *Phoma* sp. in a stirred-tank bioreactor

**DOI:** 10.1007/s13205-016-0557-9

**Published:** 2016-10-27

**Authors:** Thiarles Brun, Jéssica E. Rabuske, Izelmar Todero, Thiago C. Almeida, Jair J. D. Junior, Gustavo Ariotti, Tássia Confortin, Jonas A. Arnemann, Raquel C. Kuhn, Jerson V. C. Guedes, Marcio A. Mazutti

**Affiliations:** 1 Department of Chemical Engineering, Federal University of Santa Maria, Av. Roraima, 1000, Santa Maria, RS 97105-900 Brazil; 2 Department of Crop Protection, Federal University of Santa Maria, Av. Roraima, 1000, Santa Maria, RS 97105-900 Brazil

**Keywords:** Microorganisms, Secondary metabolites, Fermentation, Bioreactors

## Abstract

The objective of this work was to produce an herbicide by submerged fermentation in a stirred-tank bioreactor and to assess the potential herbicidal in pre-emergence, post-emergence, and in a detached leaves of *Cucumis sativus* var species. wisconsin (cucumber) and *Sorghum bicolor* (sorghum) species. Fermentations were carried out in a stirred-tank bioreactor with useful volume of 3L. Stirring rate (40, 50, and 60 rpm) and aeration (1, 2 and 3 vvm) were the variables studied for bioherbicide production. Fermented broth was fractioned with different solvents to identify the molecules produced by the fungus in a multi-dimensional gas chromatograph system. Bioherbicide showed 100% inhibition of germination of both species in the pre-emergence tests. From detached leaves tests were verified yellowish lesions in *Cucumis sativus* and necrotic lesions on leaves of *Sorghum bicolor*. Post-emergence test presented variation of the phytotoxicity from 25 to 66% for the species *C. sativus* and from 32 to 58% by *S. bicolor*. The metabolites produced by submerged fermentation of *Phoma* sp. presented activity in pre-emergence, post-emergence, and detached leaves of *C. sativus* and *S. bicolor* and it could be an alternative in the future for weed control.

## Introduction

In recent years, the market for organic foods is increasing as well as the concept of sustainable agriculture. The development of safe and eco-friendly herbicides is an emergent necessity to control weeds in these cultivations (Yang et al. 2014). Biological weed control strategies can potentially address this need and provide novel modes of action that will inhibit the growth of weeds that are resistant to more commonly used herbicides (Harding and Raizada 2015). Inundative biological control (which refers to the application of propagation of fungal spores or bacterial suspensions in concentrations that would not normally occur in nature with the intention of destroying a pest species within a managed area) is the strategy more employed (Bailey et al. 2011).

Although, a great number of microbial herbicide has been developed, only a few of them are available in commercial forms due to several constraints in the formulation, application, and commercialization. Biocontrol agents probably fail to be marketed internationally as these are living organisms and are fearful to introduce them from foreign countries (Chutia et al. 2007). For this reason, the future trend is the production of herbicidal compounds by fermentation, extract it from fermented broth, and use this compound in a more stable formulation. This strategy will not be limited on the continued survival of a given organism in an uncontrolled environment (Harding and Raizada 2015).

The use of microbial metabolites as bioherbicide is well reported in literature, especially for fungus of genera Phoma (Hubbard et al. 2015, 2016; Kalam et al. 2014; Bailey et al. 2013; Andolfi et al. 2013). In a previous study, we also have used *Phoma* sp. to produce a bioherbicide by solid-state fermentation (Klaic et al. 2016), with promising results. However, the scale-up of solid-state fermentation process is complicated due to difficult to remove the metabolic heat generated during the process (Mazutti et al. 2010). One possibility is the production of bioherbicide by submerged fermentation, which is a process well established industrially. However, it is necessary to evaluate the influence of process variables such as stirring rate and agitation on metabolite production, because biosynthesis of active secondary metabolites by fungi occurs as a specific response to the different growing environments (Bracarense and Takahashi 2014). It is well documented that stirring rate affects severally the growth of microorganism due to shear stress (Serrano-Carreón et al. 2015; Garcia-Ochoa et al. 2013; Maldonado et al. 2012). So, it is necessary to select appropriate levels for these variables in bench-scale bioreactors before scale-up the process (Formentini et al. 2014).

Based on these aspects, the present study investigates the bioherbicide production by *Phoma* sp. in a stirred-tank bioreactor containing liquid media. Stirring rate (40, 50, and 60 rpm) and aeration (1, 2 and 3 vvm) were the variables studied. Different bioassays were applied to assess the herbicidal activity of fermented broth without cells in pre-emergence, post-emergence, and in detached leaves of *C. sativus* and *S. bicolor*. In the assay with maximum herbicidal activity, the molecules produced were fractioned with different solvents and further identified in a multi-dimensional gas chromatograph system.

## Materials and methods

### Materials

Corn steep liquor (CSL) was obtained from Ingredion (Mogi Guaçu, SP, Brazil) and sucrose (Cristal) was purchased in a local market. All other chemicals, namely, (NH_4_)_2_SO_4_, FeSO_4_·7H_2_O, MnSO_4_·H_2_O, and MgSO_4_, were purchased from Sigma-Aldrich.

### Microorganism, inoculum, and fermentations

The strain of *Phoma* sp. (NRRL 43879) was obtained at the National Center for Agricultural Utilization Research—EUA (ARS). It was maintained in a potato dextrose agar (PDA) at 4 °C and subcultured every 15 days. Cell production for inoculum was incubated in a Petri dish containing PDA for 8 days at 28 °C. Afterwards, a disk of 6 mm of fungal mycelium was transferred to an Erlenmeyer flask containing 100 mL of fermentation medium at 28 °C, 120 rpm for 5 days (Innova 44R, NewBrunswick) for inoculum.

The fermentations were carried out in a batch bioreactor (BIOTEC-C, Tecnal, Brazil), containing 3.0 L of the culture medium. Fermentations were started using 10% (V/V) of inoculum at an initial pH of 6.0, 28 °C for 7 days. The fermentation medium was composed of corn steep liquor (10% v/v), sucrose (20 g L^−1^), (NH_4_)_2_SO_4_ (2 g L^−1^), MgSO_4_·7H_2_O (0.5 g L^−1^), FeSO_4_·7H_2_0 (1 g L^−1^), and MnSO_4_·H_2_O (1 g L^−1^). Biomass was separated from fermentation broth by filtration using filter paper (Whatman number 2) followed by centrifugation (Eppendorf, model 5804R) at 10,000 rpm for 10 min and the supernatant was used in the bioassays.

The effect of aeration and stirring rate on herbicidal activity was evaluated by means of a central composite design (CCD) with four assays plus three central points. The range of variables investigated was 40–60 rpm and 1–3 vvm and the range was defined on preliminary tests. Each fermentation was considered as one treatment in the bioassay. The responses evaluated were the inhibition of germination in pre-emergence, phytotoxicity in post-emergence, and in the punctured detached leaves.

### Bioassays

Three different bioassays were carried out to investigate the herbicidal activity of fermented broth without cells: pre-emergence, post-emergence, and detached leaves of *Cucumis sativus* var. Wisconsin and *Sorghum bicolor*.

### Pre-emergence

The germination tests were carried by applying 10 mL of fermented broth without the cells in a germitest paper containing 25 seeds of each culture and maintained at 25 °C (POL-EKO, model KK 350). Control tests were carried out replacing fermented broth by culture media and distilled water. The seed germination was evaluated at 4 (first count) and 10 (second count) days after the application of bioherbicide, according to Brazilian rules for seed analysis (Brasil 2009). Afterwards, the germinated or non-germinated seeds were counted. All seeds that presented the primary root protrusion higher than 2 mm were considered as germinated. Each treatment was replicated four times.

### Punctured leaf assay

The phytotoxic effects of the fermented broth without cells were assessed by a punctured leaf assay. Well-expanded leaves of 2-week-old *C. sativus* e *S. bicolor* plants grown in a greenhouse were punctured (base, summit, and center) using a sterile fine-pointed needle, and droplets (1 µL) of each treatment solution were deposited on each of these sites (2 punctures per leaf, 3 leaves per solution). After droplet application, the plants were maintained at 25 °C (POL-EKO, model KK 350) with photoperiod of 12 h, and the diameter of the lesions was measured after 72 h.

### Post-emergence

The herbicidal activity was determined using *C. sativus* and *S. bicolor* as target plants. A completely randomized design composed of eight treatments (each fermentation of DCC + a control) and four repetitions, where each repetition was represented for a tray containing four plastic cups with volume of 200 mL with commercial substrate (Macplant^®^) without any treatment. Three seeds were sown in each vessel, and after the emergence, only one plant was maintained in each vessel and cultivated for 7 days in a greenhouse located at the Federal University of Santa Maria (Santa Maria, Brazil).

A volume of 50 mL of fermented broth was applied at the same time in each bioassay using a garden sprayer. Control assays were performed using the culture medium instead fermented broth, and also using distilled water. Seven days after the application, plant injury was visually estimated as percent growth reduction by comparing to untreated controls, where 100% represents complete plant death and 0% represents no effect (Frans and Crowley, 1986). In addition, we investigated other factors such as (1) height of plants; (2) fresh weight of aerial and root parts; and (3) dry weight of aerial and root parts.

### Liquid–liquid extraction of molecules

Liquid–liquid extractions were carried out mixing 50 mL of fermented broth without cells with 50 mL of organic solvents with different polarities (methanol, ethanol, and ethyl acetate) in an equilibrium jacketed glass cells (100 mL). The extractions were carried out at 10 °C using a thermostatic bath (Quimis, Brazil) under magnetic stirring (Ika Werke, model RH–KT/C, Staufen, Germany) to promote agitation of the mixture during 12 h. After this procedure, the phases were separated and the organic solvent fraction was used for chemical identification of the compounds in a gas chromatograph.

### Identification of molecules by multi-dimensional gas chromatograph system

The molecules produced by the fungus were determined using a multi-dimensional gas chromatograph system (Shimadzu, model MDGC/GCMS-2010) equipped with a mass spectrometer detector (QP-2010 Ultra) and flame ionization detector (FID-2010 Plus), and automatic injection system (AOC-20i). A volume of 1 µL of samples was injected in split mode (30:1) at 250 °C. The separation of compounds was carried out in a capillary column Rtx-1 MS (30 m × 0.32 mm × 0.1 µm). Helium was the carrier gas with a flow rate of 2 mL min^−1^ at a pressure of 5.05 psi; electronic impact mode of 70 eV. Initial temperature of the column was set at 70 °C which was increased up to 250 °C at a rate of 3 °C min^−1^ and kept isothermal for 10 min. The temperature of detector was set at 250 °C. The compounds were identified by comparison of the mass spectrum with those of database (Wiley, 9° Edition).

### Statistical analysis

All statistical analysis were performed using Statistica 8.0 software (Statsoft Inc., Tulsa, OK, USA), considering a 95% significance level. Statistical differences between treatments were determined by one-way analysis of variance and means separated using the least significant difference test (*p* < 0.05).

## Results and discussion

Table 1 presents the results obtained in the bioassays with *C. sativus* and *S. bicolor* for all runs of CCD (T1-T7) and also for the control treatment (T0). In the pre-emergence, all treatments of CCD showed inhibitory effect on germination of *C. sativus* and *S. bicolor* in the first and second count, where in the second count the inhibition of germination was 100% for all treatments of CCD. On the other hand, the control treatment presented 100% of germination for the two species. In the first count, the treatment that caused the largest interference in the germination of *C. sativus* was T4, with 40% inhibition of germination, whereas for *S. bicolor* it was T6, with 84% inhibition. The metabolites produced by *Phoma* sp. have a broad herbicidal action spectrum in pre-emergence, since they have effect on the group of monocotyledonous (*S. bicolor*) and dicotyledonous plants (*C. sativus*). This result is slightly different than those reported by Bailey et al. (2011), which produced a bioherbicide with *Phoma* sp. and obtained a better effect on dicotyledonous seeds than in monocotyledons. The variables investigated did not affect the herbicidal activity in pre-emergence, since total inhibition was obtained for both species independent of stirring and aeration rates employed.

**Table 1:** Influence of process variables on the inhibitory effect of fermented broth of *Phoma* sp. in the different bioassay

Treatments	Stirring rate (rpm)	Aeration rate (vvm)	Pre-emergence				Punctured leaf		Post-emergence	
			*C. sativus*		*S. bicolor*		*C. sativus*	*S. bicolor*	*C. sativus*	*S. bicolor*
			1° count (%)	2° count (%)	1° count (%)	2° count (%)				
T0	–	–	0^ba^	0^b^	0^c^	0^b^	N*	N	0d^b^	0^c^
T1	40 (−1)	1 (−1)	12^a^	100^a^	55^b^	100^a^	–	+	49.4^b^	58.1^a^
T2	60 (1)	1 (−1)	24^a^	100^a^	81^a^	100^a^	–	+	25.0^c^	43.1^b^
T3	40 (−1)	3 (1)	29^a^	100^a^	61^b^	100^a^	–	+	66.9^a^	41.2^b^
T4	60 (1)	3 (1)	40^a^	100^a^	72^a^	100^a^	–	++	65.0^a^	58.1^a^
T5	50 (0)	2 (0)	16^a^	100^a^	51^b^	100^a^	–	+	61.2^a^	32.5^b^
T6	50 (0)	2 (0)	12^a^	100^a^	84^a^	100^a^	–	+	56.9^a^	40.6^b^
T7	50 (0)	2 (0)	26^a^	100^a^	80^a^	100^a^	–	+	45.6^b^	39.4^b^

^a, b^Different letters in the column represent a significant difference at 95% (*p* < 0.05-Tukey test)

* N without effect, − light chlorosis, – sharp chlorosis, + light necrosis, ++ sharp necrosis

Results for punctured leaf assay demonstrated the occurrence of injuries as mild yellowing and light necrosis in detached leaves of *C. sativus* and *S. bicolor*, respectively. These spots were observed on the first day after the evaluation and intensified until the last evaluation, which was performed 72 h after the treatment application. The most pronounced damage was found in the treatment T3 for *C. sativus* and T4 for *S. bicolor*, showing severe chlorosis of leaves of *C. sativus* and severe necrosis for *S. bicolor*. The injury verified in our work for the punctured leaf assay is in good agreement with other studies for Phoma species. Vikrant et al. (2006) applied filtered broth of *Phoma herbarum* obtained by submerged fermentation on detached leaves of *Parthenium hysterophorus* (ragweed) and verified fast yellowing followed by necrosis and death of the leaves 48 h after treatment. Using a puncture test, made with leaves of *Stellaria media*, *Urtica dioica*, *Sonchus arvensis*, *Parietaria officinalis*, *Lactuca serriola,* and *Helianthus annuus,* the metabolites produced by the fungus *Phoma chenopodiicola* caused chlorosis and necrosis in the leaves of these species (Evidente et al. 2015).

The results obtained in punctured detached leaves of *C. sativus* and *S. bicolor* showed the incidence of chlorosis or necrotic lesions, but with low intensity. So the detached leaf bioassay was not considered significant to choose the best condition for production of metabolites with herbicidal action. The low effect verified may be related to the low concentration of metabolites present in fermented broth without the addition of adjuvant to improve the efficacy. Varejão et al. (2013) reported that the phytotoxins are often present in low concentrations in the filtrate coming from the fermentation processes of microorganisms.

**Fig. 1:**
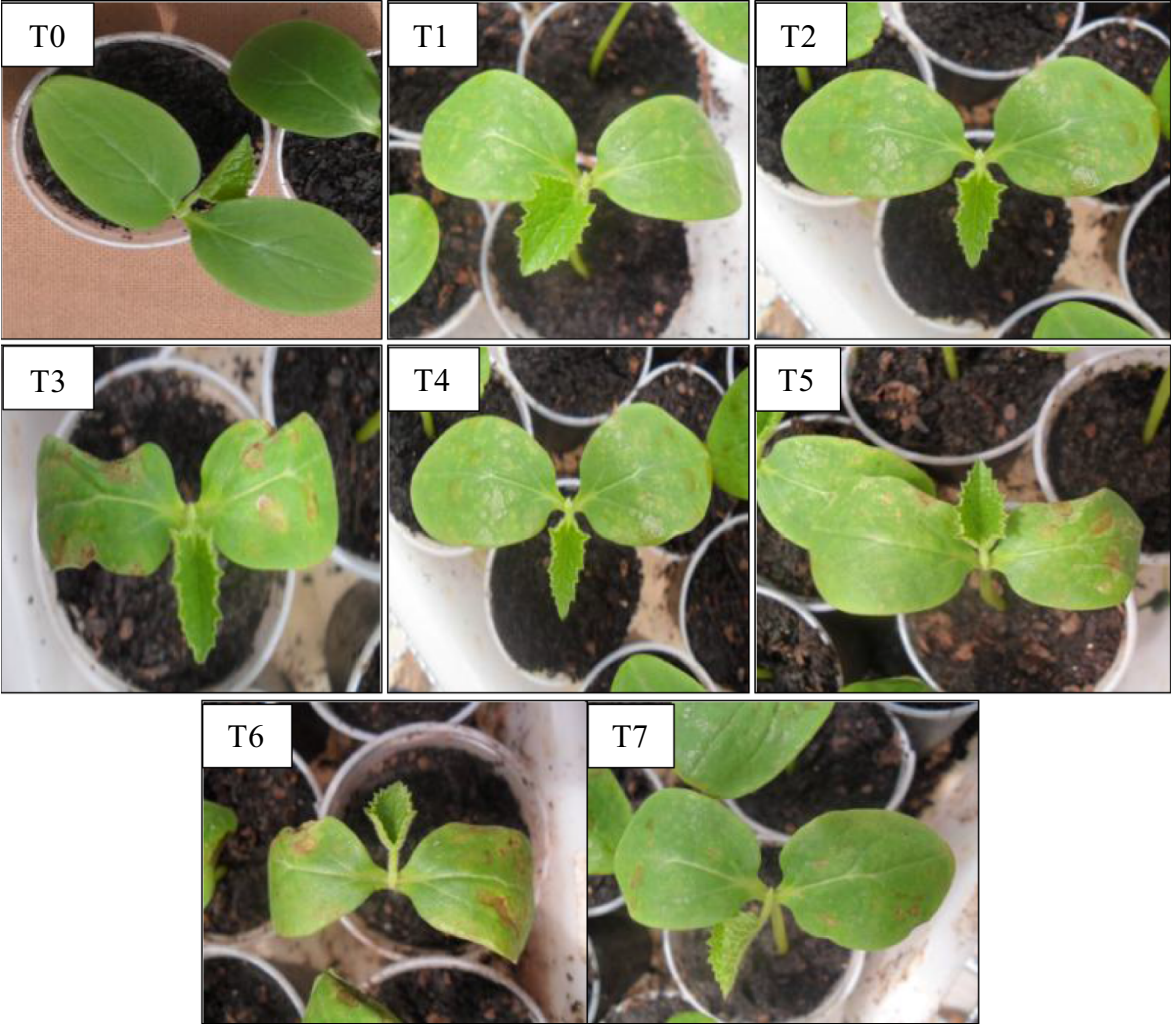
Photograph illustrating the lesions caused by the fermented broth of *Phoma* sp. in *C. sativus*

**Fig. 2:**
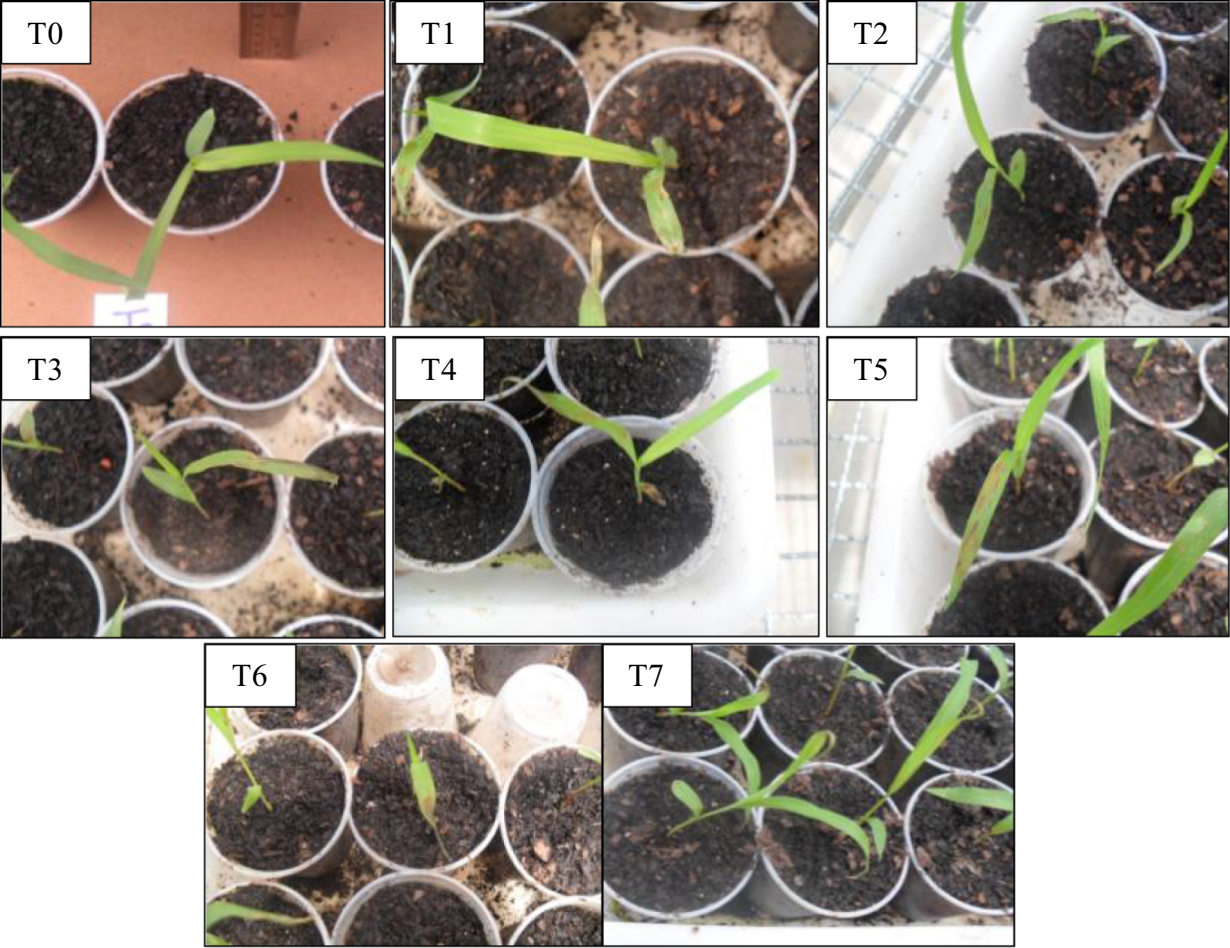
Photograph illustrating the lesions caused by the fermented broth of *Phoma* sp. in *S. bicolor*

For post-emergence bioassays, the treatments that presented the highest percentage of phytotoxicity for *C. sativus* were T3 (66.8%), T4 (65.0%), T5 (61.2%), and T6 (56.8%), while for *S. bicolor* the best results were found in T1 (58.1%) and T4 (58.1%). In these treatments there are no statistical difference according to the Scott-Knott test (*p* < 0.05).Although no significant statistical differences among treatments were found, the greatest potential phytotoxic on the species assessed was T3 and T4 for *C. sativus* and *S. bicolor*, respectively (Figs. 1, 2). The plants for the control test did not have phytotoxicity, demonstrating that the injury is due to the presence of compound (or compounds) in the fermented broth. Figure 1 and 2 show the damage caused by the application of fermented broth of *Phoma* sp. on the leaves ranged from a slight chlorosis until wilted appearance or necrotic lesions. Vikrant et al. (2006) applied the filtered broth of *Phoma herbarum* and observed damage such as yellowing, followed by sharp withers and complete collapse of seedlings *Parthenium hysterophorus* (ragweed). Cimmino et al. (2013) tested the potential of chenopodolin metabolite produced by the fungus *Phoma chenopodicola* in *Cirsium arvense* and *Setaria viride* (monocotyledon and dicotyledonous, respectively), verifying injuries as necrosis, wilting, and tissue degradation in general.

**Fig. 3:**
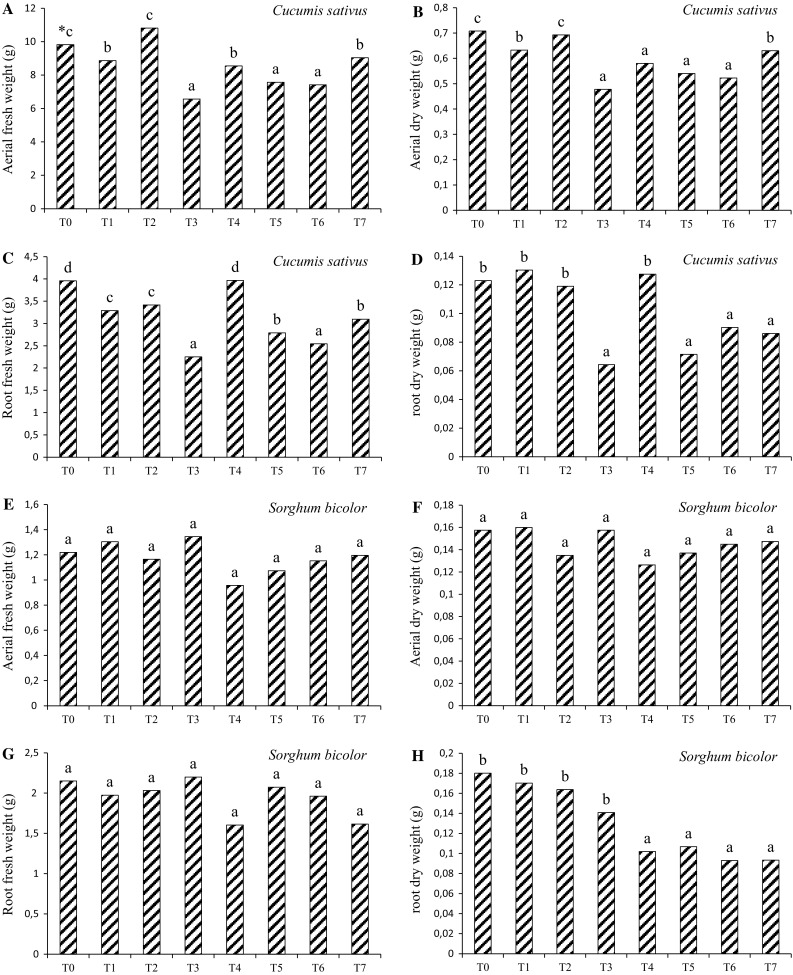
Fresh and dry weight of aerial and root parts of *C. sativus* and *S. bicolor* obtained in the treatments. *Different letters* represent a significant difference at 95% (*p* < 0.05-Tukey test)

**Fig. 4:**
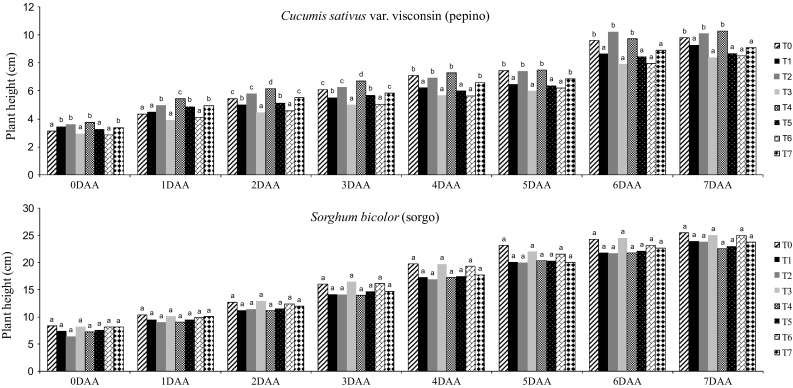
Height of plants evaluated daily during 7 days. *Different letters* represent a significant difference at 95% (*p* < 0.05-Tukey test)

The fresh and dry weight of aerial and root parts of plants at 7 days after the application of fermented broth are showed in Fig. 3. For *C. sativus,* the treatment T3 reduced fresh and dry weight of aerial and root parts. For *S. bicolor*, the highest inhibitory effect was observed in T4, reducing also the fresh and dry weight of aerial and root parts. Figure 4 presents the results of referring to variation of plant height among the treatments assessed daily during 7 days after application. No significant difference between T3 and T6 treatments were observed for *C. sativus*. However, the highest herbicidal effect was observed in T3, causing a reduction in plant height. For *S. bicolor*, no significant difference (*p* < 0.05) between treatments also was observed, however, the treatment T4 showed the highest reduction in the growth.

The best results were obtained in T3 for *C. sativus* and T4 for *S. bicolor*. The difference in T3 and T4 was the stirring rate (40 and 60 rpm, respectively) both at 3 vvm. Modification of process variables as stirring rate may affect the excretion of different compounds in the media, because biosynthesis of active secondary metabolites by fungi occurs as a specific response to the different growing environments (Bracarense and Takahashi, 2014). This is corroborated by comparing the chemical composition of T3 and T4 experiments (Tables 2, 3, respectively), where the profile and the concentration (expressed as normalized peak area) were different.

**Table 2:** Chemical profile obtained in treatment T3

	Compound	Chemical structure	RT	Área (ua)	% Normalized area
Methanol					
1	2-Oxiraneethanol, 2-t-butyldimethysilyloxymethyl- acetate	C13H26O4Si	9.077	24,551	1.088
2	Acetate, [3-(acetyloxy)-4,5-dihydro-5-isoxazolyl]methyl	C8H11NO5	11.117	19,396	0.86
3	1,4-Diacetyl-3-acetoxymethyl-2,5-methylene-l-rhamnitol	C14H22O8	11.485	186,646	8.273
4	Uric acid	C5H4N4O3	11.518	15,193	0.673
5	Spermine	C10H26N4	11.548	10,497	0.465
6	12-Methyl-oxa-cyclododecan-2-one	C12H22O2	11.594	16,766	0.743
7	3,7-Diazabicyclo[3.3.1]nonane, 9,9-dimethyl-	C9H18N2	11.778	128,511	5.696
9	Pyrrolo[1,2-a]pyrazine-1,4-dione, hexahydro-3-(2-methylpropyl)-	C11H18N2O2	12.547	1,454,291	64.463
10	12-Dimethylamino-10-oxododecanoic acid	C14H27 NO3	12.666	400,130	17.736
Ethanol					
1	Methyl 4,6-decadienyl ether	C11H20O	11.147	6864	0.548
2	3-Trifluoroacetoxydodecane	C14H25F3O2	11.316	26,446	2.11
3	5-Dodecanol acetate	C14H28O2	11.58	30,363	2.423
4	Pyrrolo[1,2-a]pyrazine-1,4-dione, hexahydro-	C7H10N2O2	11.612	64,921	5.18
5	3,7-Diazabicyclo[3.3.1]nonane, 9,9-dimethyl-	C9H18N2	11.809	21,095	1.683
6	Pyrrolo[1,2-a]pyrazine-1,4-dione, hexahydro-3-(2-methylpropyl)-	C11H18N2O2	12.559	1,103,547	88.056
Ethyl acetate					
1	Pyrrolo[1,2-a]pyrazine-1,4-dione, hexahydro-3-(2-methylpropyl)-	C11H18N2O3	12.699	18,577	43.020
2	2,2-Dipropyl-N-ethylpiperidine	C13H27N	12.778	15,358	35.564

*RT* retention time

**Table 3:** Chemical profile obtained in treatment T4

	Compound	Chemical structure	RT	Área (ua)	% Normalized area
Methanol					
1	3-Trifluoroacetoxydodecane	C14H25F3O2	11.1	10,502	0.01
2	Pyrrolo[1,2-a]pyrazine-1,4-dione, hexahydro-	C7H10N2O2	11.4	129,668	0.19
3	Pyrrolo[1,2-a]pyrazine-1,4-dione, hexahydro-3-(2-methylpropyl)-	C11H18N2O2	12.5	1,278,230	1.89
4	Hydroquinine, 2′-propoxy-	C23H32N2O3	18.6	5,712,157	84.44
5	Acetic acid, 3-acetoxy-5-pentyl-2-(4,6,6-trimethylbicyclo[3.1.]	C25H34O4	22.3	9,104,318	13.45
Ethanol					
1	Uric acid	C5H4N4O4	11.7	699,460	19.92
2	Pyrrolo[1,2-a]pyrazine-1,4-dione, hexahydro-	C7H10N2O2	12.4	2,328,303	66.31
3	1-Undecanethiol	C11H24S	12.6	456,483	13.00
4	3,7-Diazabicyclo[3.3.1]nonane, 9,9-dimethyl-	C9H18N2	15.3	14,231	0.405
5	Pregn-4-ene-3,20-dione, 17,21-dihydroxy-, bis(O-methyloxime)	C23H36N2O4	16.1	12,601	0.359
Ethyl acetate					
1	2,4,7-Trioxabicyclo[4.4.0]dec-9-ene, 8-decyloxy-3-phenyl-	C23H34O4	12.0	7,694	33.48
2	Pyrrolo[1,2-a]pyrazine-1,4-dione, hexahydro-3-(2-methylpropyl)-	C11H18N2O2	12.7	11,368	49.46

*RT* retention time

Some compounds were found more frequently in both analyzed fractions, highlighting the pyrrolo [1,2-a] pyrazine-1,4-dion, Hexahydro-3-(2-methylpropyl) that were observed in greater abundance for most of the analyzed fractions, always having the largest peak area. These compounds have similarities with two other phytotoxic metabolites from *Alternaria alternata* with herbicidal activity, namely, Maculosin-1 {(I) (3S-cis) -3-hexahydro- [(4-hydroxyphenyl) methyl] pyrrolo [1,2-a] pyrazine -1,4-dione}, and Maculosin-2 {(II) (3S-cis) hexahydro-3-methyl-phenyl pyrrolo [1,2-a] pyrazine-1,4-dione} (Bobylev et al. 2000). An herbicide which has a pyrazine cycle in its molecule is the Diquat dibromide. Melo et al. (2014) fractioned fermented broth of *Mortierella alpine* with different organic solvents and identified the presence of chemical class of alkaloids pyrrolopyrazine: (a) pyrrolo [1,2-a] pyrazine-1,4-dione, hexahydro-3- (2 methylpropyl) and (b) pyrrolo [1,2-a] pyrazine-1,4-dione, hexahydro-3- (phenylmethyl). Other studies indicate that alkaloids as pyridine pyrrole occurs in many endophytes. These compounds have been isolated from species *Neotyphodium* and *Epichloe* spp. which are grasses in cold regions, which are used to protect plants against worms and plant pathogens (Nan and Li, 2000; Malinowski et al. 2005; Zhang et al. 2012).

Based on the literature, it is possible that the compound called pyrrolo [1,2-a] pyrazine-1,4-dione, hexahydro-3- (2-methylpropyl) presents herbicidal effect on monocotyledons and dicotyledonous plants, but more studies are required to confirm this statement. The highest abundance of this compound was found in T3 where the agitation conditions were minimal (40 rpm) and the highest aeration rate (3 vvm). These results may be related to the oxygen requirement by fungi provided by aeration allowing greater production of the compound of interest. On the other hand, the stirring rate was not considered a limiting factor for the production of these compounds in the studied range, showing that a minimal agitation was sufficient to homogenize the culture medium without negatively impacting the microbial growth.

## Conclusions

The highest herbicidal activity of fermented broth of *Phoma* sp. was obtained in T3 for *C. sativus* and T4 for *S. bicolor*. In these runs, stirring rate was 40 rpm and 60 rpm, respectively, whereas aeration rate was 3 vvm. The compound pyrrolo [1,2-a] pyrazine-1,4-dione, hexahydro-3- (2-methylpropyl), which is reported in the literature as having herbicidal effect, may be the main metabolite produced by *Phoma* sp. Secondary metabolites produced by *Phoma* sp. have herbicidal action on pre-emergence, post-emergence and punctured detached leaves of *C. sativus* and *S. bicolor*, and may become an alternative in the future for weed control.
